# The effect of rearing conditions during the milk-fed period on milk yield, growth, and maze behaviour of dairy cows during their first lactation

**DOI:** 10.5194/aab-64-69-2021

**Published:** 2021-02-23

**Authors:** Jan Broucek, Michal Uhrincat, Peter Kisac, Anton Hanus

**Affiliations:** National Agricultural and Food Centre, Research Institute of Animal Production Nitra, Hlohovecka 2, 951 41 Luzianky, Slovakia

## Abstract

The objective was to find whether cow growth, milk performance, and behaviour are affected by (1) rearing conditions until weaning after a milk-fed period of 84 d and (2) the sire lineage. Thirty-five Holstein heifers were assigned to one of three treatments: SM, n=13, pen with mother to 21st day, then group pen (they received a maximum of 6 kg of milk daily); SN, n=9, after 3 d with own mother in pen with nursing cow (they received a maximum of 6 kg of milk daily); H, n=13, in hutch from the 2nd to 56th day (6 kg of milk replacer daily), then loose housing pen to weaning (6 kg of milk replacer daily). After weaning at the 84th day, all heifers were kept in pens with the same ration as during calving. During lactation, live body weight (LBW) was measured each month and milk yield each day. Maze learning was evaluated in the fifth month of lactation. The data were analysed using a general linear model ANOVA. At the 30th day, the LBW tended to be the highest in SN (SM 528.2 ± 11.4 kg, SN 571.7 ± 15.3 kg, H 533.2 ± 12.3 kg). When lactation ended, the highest LBW was in SN and the lowest in H (SM 612.6 ± 12.2 kg, SN 623.1 ± 16.4 kg, H 569.8 ± 13.2 kg; P<0.05). The SN tended to have the highest production of milk (SM 7143.9 ± 241.5 kg, SN 7345.1 ± 319.0 kg, H 7146.7 ± 234 kg), and the H for FCM (SM 6290.3 ± 203.2 kg, SN 6307.6 ± 268.4 kg, H 6399.3 ± 197.1 kg) for 305 d lactation. Group SN crossed the maze fastest (SM 1141.4 ± 120.5 s, SN 810.3 ± 160.5 s, H 1120.8 ± 118.6 s). The vocalization number differed significantly (SM 32.3 ± 5.7, SN 20.8 ± 4.4, H 9.9 ± 2.6; P<0.01). The results indicated that the rearing method up to weaning may have an impact on dairy cows' performance and behaviour.

23 February 2021

## 1 Introduction

Milk and milk replacer (MR) feeding strategies have been studied for many years. Currently, this issue is gaining in importance in connection with the welfare of calves and dairy cows.

A number of studies have explored different ways of keeping cows and calves together and examined possible benefits of this more natural rearing system (Loberg and Lidfors, 2001; Wagenaar and Langhout, 2007; Loberg et al., 2008). Suckling systems are more beneficial to the welfare of calves than the more common artificial rearing systems (Krohn, 2001; Mala et al., 2019). Contact with older animals during the first few weeks of life is known to stimulate calves to consume more rough feeds, especially before weaning. Increased feed intake manifests itself even later (Albright and Arave, 1997; Loberg et al., 2008; Costa et al., 2016). Group housing with animals of the same age may also stimulate appetite (Yanar et al., 2000; Hepola et al., 2006; Wójcik et al., 2013). The majority of studies have reported that the benefits for growth during the suckling period, compared with separated calves, persisted for up to 16 months (Flower and Weary, 2003; Khan et al., 2011; Meagher et al., 2019).

Bovines are highly motivated for social contact. Krohn et al. (1999) concluded that social interaction between cow and calf in the colostrum period and with other calves had a positive effect on the daily gain of the calf. The mother–calf bond may have positive effects on behaviour development and the learning capabilities of calves (Rushen and de Passillé, 1998; Loberg et al., 2008; Steele, 2019). The social housing in the group pen increased concentrate intake during the pre-weaning period, resulting in greater weight gains after weaning. The group housing has space enough for calves to exercise and play (Valnickova et al., 2015; Johnsen et al., 2016). Both social facilitation and social learning may result in socially housed calves showing higher intakes of solid feed and improved live body gains compared with individually housed calves (Paula Vieira de et al., 2012; Costa et al., 2015). According to Costa et al. (2016), calves raised in isolation (hutch) exhibit deficient social skills, difficulties in coping with novel situations, and poor learning abilities.

In animal husbandry it is common practice to separate a dairy cow and her calf shortly after birth, but this practice is debated because of animal welfare concerns. Early weaning has been shown to affect normal behavioural development and compromise the animal's ability to cope behaviourally with later challenges of environment conditions (Rushen and de Passillé, 1998). Under natural conditions, the cow and calf remain together until weaning at 6–8 months (Krohn, 2001). In contrast, on many commercial dairy farms, calves are separated from cows within a few hours of birth (Flower and Weary, 2003).

Abrupt weaning from milk at the same time as breaking the social bond with the mother is a known stressor (Meagher et al., 2019; Wagenaar and Langhout, 2007). According to Khattak et al. (2018) the weaning at a later age (70, 90, or 110 d) might contribute significantly to the feed intake and body weight gain of calves.

It has been formulated that intensive growth programmes for dairy heifers could lead to increased milk production in later life. Several studies suggest that a pre-weaning calf's weight gains and high live body weight (LBW) for heifers at calving had a positive effect on milk production in the first lactation (Bar-Peled et al., 1997; Langhout and Wagenaar, 2006; Terré et al., 2009; Johnsen et al., 2016).

However, we must distinguish between milk feeding and milk replacer (MR). Milk is more important in terms of welfare (Krohn, 2001; Guler et al., 2003; Langhout and Wagenaar, 2006; Johnsen et al., 2016) and later performance of heifers (Shamay et al., 2005; Moallem et al., 2010). But even heifers fed more intensively (increased MR amount or crude protein content) until weaning achieve an increased milk yield during the first lactation (Ballard et al., 2005; Drackley et al., 2008).

The use of modern housing systems needs milking cows resistant to stress and able to adapt to altered conditions of the environment in coherence with new procedures and methods of management (robotic feeding and milking). Learning has been defined as a relatively permanent change in response over time as a result of practice or experience (Kilgour, 1987). The speed and correctness of an animal in running through various types of mazes was used as a measure of learning ability for a long time (Kilgour, 1987; Stewart et al., 1992; Arave, 1996; Fraser and Broom, 1997). The ability to learn allows the individual animal to adapt behaviourally to changes in its environment (Kilgour, 1981; Albright and Arave, 1997; Broom and Fraser, 2007). Cows are able to learn to traverse a complex maze when they are provided with step-by-step learning opportunities (Wredle et al., 2004). A discrimination learning task with cattle found that high milk producers learn more rapidly than low producers (Kilgour, 1987). Hirata et al. (2016) showed that the ability of cows to learn was limited to about 20 % of animals.

The objective was to find whether cow growth, milk performance, and behaviour are affected by rearing conditions until weaning at 84 d and the sire lineage.

## 2 Material and methods

### 2.1 Ethical statement

The authors declare that the experiments comply with the current laws of the Slovak Republic. The treatment of the animals was approved by the Ministry of Agriculture and Rural Development of the Slovak Republic (no. 115/1995 Z.z. and 377/2012 Z.z.). The experiments were carried out in accordance with the Code of Ethics of the EU Directive 2010/63/EU for animal experiments.

### 2.2 Treatment

At birth, 35 Holstein heifers (descended from four sires) were randomly assigned to one of three rearing treatments:

- Group SM (n=13) was made up of heifers separated in an individual pen with their mother until the 21st day (milked from the second day); they were suckled at the mother's udder for 10 min three times per day (08:00, 13:00, 18:00 LT). They received a maximum of 6 kg of whole milk per day. The calf was separated in a pen of 1.2 × 4.5 m. Then calves were kept in a loose housing pen from the 22nd day (6 kg of whole milk per day, twice daily 3 kg, bucket with drinking nipple). SM calves were weighted before and after each suckling. Suckling time a mother's udder (three times 10 min) was determined during the preparation of the experiment according to Passillé de and Rushen (2006).
- Group SN (n=9) was made up of calves who spent 3 d with their own mother in individual pens and then moved to a pen with nursing cows from the fourth day; calves could suckle at any time (they received a maximum of 6 kg of whole milk per day). Calves had to compete to suckle at the nursing cow.
- Group H (n=13) consisted of calves who, after having been nursed by their dams in individual pens for 24 h, moved to hutches from the 2nd to the 56th day (bucket with drinking nipple, MR, 2nd day three times 0.5 kg, 3rd day three times 1.0 kg, 4th day three times 1.5 kg, from the 5th day to the 21st day 6 kg per day, three times daily) and then to a loose housing pen to be weaned from the 22nd day (bucket with nipple, MR, 6 kg per day, twice daily).

From the 4th to the 84th day, the heifers of the SM group had an intake of 407.15 ± 10.73 kg (5.09 kg/d) milk, the SN group had 414.02 ± 8.92 kg (5.17 kg/d) milk, and the calves of the H group had 408.12 ± 9.12 kg (5.10 kg/d) of MR. Differences were not significant. The amount of milk drunk did not increase with age; the calves just drank faster. We needed to have consumption comparable to other groups.

In summary, group SM was allowed 21 d of suckling and 63 d of bucket nipple feeding; SN was allowed 84 d of suckling; H was allowed 1 d of suckling and 83 d of bucket nipple feeding. The weaning was performed abruptly without decreasing the milk allowance. This is common practice (Vasseur et al., 2010; Scoley et al., 2019). The majority of Slovakian farmers operate an abrupt weaning strategy. The weaning was also performed on Saturdays and Sundays. Every day, one of the authors and a technician were present.

All animals were weaned at the age of 12 weeks and moved to a group housing pen, where equal conditions of nutrition were ensured. The transfer was made at the exact age of 84 d. Each treatment group had its pens; pens were also differentiated by age. The principle was observed that the age difference in one pen was not higher than 21 d. The live body weights (LBWs) at weaning were as follows: SM, 97.0 ± 4.3 kg; SN, 104.5 ± 4.6 kg; H, 79.1 ± 3.1 kg; P=0.0023.

Experimental calves originated from four sires (S1, S2, S3, and S4). The distribution was as follows: SM – S1: 3; S2: 2; S3: 3; S4: 5; SN – S1: 0; S2: 5; S3: 3; S4: 1; H – S1: 1; S2: 3; S3: 7; S4: 2.

After weaning from milk feeding (at the 84th day), all heifer calves were kept in age-balanced groups in loose housing bedded pens with the same ration as to calving. Approximately 15 heifers were kept in a pen of 9 × 4.5 m. Feed was available throughout the 24 h periods. Heifers were fed alfalfa hay and corn silage ad libitum and 1.5 kg concentrate per day after weaning. The concentrate mixture (JKS, PZa Slovakia, dry matter (DM) 90.1 %) contained sunflower cake, cotton seed cake, corn, wheat bran, mineral mixture, and salt (crude protein 183 g/kg DM, crude fat 35 g/kg DM, and ash 92 g/kg DM).

The breeding programme of heifers began at 13 months of age; the limiting live body weight for a breeding age was 360 kg. Heifers were bred by artificial insemination (AI) with frozen–thawed semen. Hormonal breeding programmes were not used. Confirmation of pregnancy was performed by palpation per rectum 6–8 weeks after insemination. All inseminations and pregnancy diagnoses were done by the same operator.

The ages of the first service interval and the conception were as follows: SM, 432.3 ± 4.8 d; SN, 423.3 ± 7.6 d; H, 445.1 ± 4.5 d; P=0.0086; SM, 457.0 ± 6.3 d; SN, 448.2 ± 9.8 d; H, 486.1 ± 4.9 d; P=0.0007. The LBWs at the first service interval and at the conception were as follows: SM, 415.6 ± 13.8 kg; SN, 402.6 ± 15.9 kg; H, 421.5 ± 11.6 kg; P=0.4000; SM, 439.2 ± 14.8 kg; SN, 430.1 ± 17.3 kg; H, 454.9 ± 14.2 kg; P=0.3169.

### 2.3 Housing and milking of heifers after calving

Cows were kept in pens (movement area 7.4 m${}^{2}$ per animal, concrete alleys 2.6 m wide) with free stalls (1.15 × 2.0 m). The groups were balanced according to lactation stage. Automatic watering troughs were located next to feed bunks and at the end of free-stall pens.

All cows were milked from the fourth day of lactation in a double-five herringbone design (with vacuum level 50 kPa, pulsation rate 55 cycles per minute and pulsation ratio 60 : 40).

Individual milk yields were recorded electronically each morning and evening milking. They were calculated as the sum of the evening and morning yields. Each electronic milk meter was checked the last day before starting the trial and then two times each week in order to calculate its deviation level. This was done by comparison of the amount of milk weighed on an electronic scale. All electronic meters had a tolerance level within 3 %.

Samples for milk composition determination were taken once per week by the milk laboratory (RIAP, Nitra) using an infrared analyser.

The cows were milked twice a day at 05:00 and 16:00 LT after being driven by the herdsman a short distance within the barn to a holding area, which measured 13.5 m × 4.5 m, adjacent to the milking parlour. Cows entered the parlour individually once a milking stall was available. Upon exiting the parlour, cows remained in a separate holding area until all other cows in the group were milked. The cows then walked through an alley and had access to their free-stall pens immediately.

### 2.4 Feeding of primiparous cows

Feed was available throughout the 24 h period, except during milking. The total mixed ration (TMR) was balanced according to Slovakian nutrient requirements for dairy cattle. The feed ration included the factors and equations adopted for maintenance, growth, reproduction, and lactation and consisted of the following stages: early lactation (first 4 months), mid-lactation (fifth to seventh month), and late lactation.

The cows were fed a TMR consisting of corn silage, alfalfa haylage, alfalfa hay, barley straw, brewer's grain, sugar-beet pulp, and concentrate mixture for high-yielding cows throughout the study. Feed ration contained 19.2 kg DM, 131.0 MJ net energy content for lactation (NEL), 1.84 kg protein digestible in the small intestine (PDI), and 2.89 kg of crude protein (early stage); 18.3 kg DM, 120.2 MJ NEL, 1.65 kg PDI, and 2.66 kg of crude protein (mid stage); 16.5 kg DM, 104.1 MJ NEL, 1.44 kg PDI, and 2.32 kg of crude protein (late stage). The total mixed diet was administered to troughs in the new cubicle barn by a feeding wagon. Feeding was allowed throughout the 24 h period, except during milking. Feed bunks were located centrally in the free-stall pens, raised 0.68 m above ground and with 0.7 m of feeding space. Cows did not receive concentrates separately.

### 2.5 Health and growth

The methods of Slavik et al. (2009) and Novak et al. (2010) for the daily evaluation of the health condition were used. During lactation, LBW was measured each month and milk yield each day. The cows were weighed on the mobile livestock scale (Deutscher Verband für Materialforschung und -prüfung e.V. (DVM), Soehnle, Germany; load capacity up to 2000 kg, weighing accuracy ± 0.2 kg).

### 2.6 Maze learning ability

**Figure 1:**
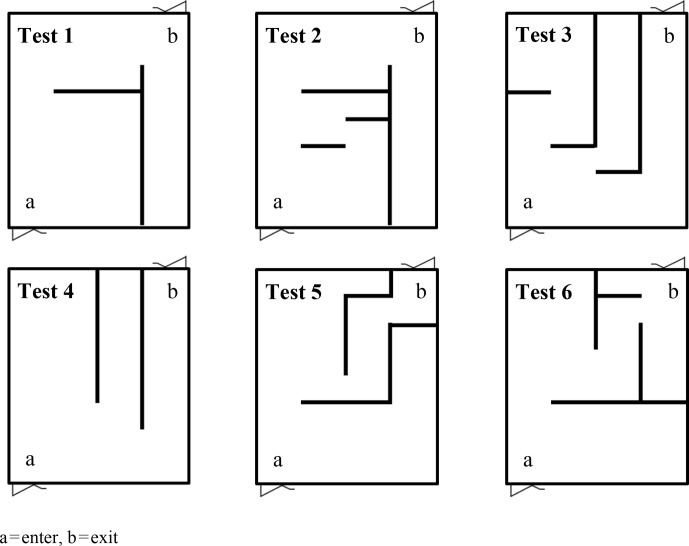
Maze learning ability tests.

Learning ability was evaluated at the age of 5 months by the Hebb–Williams test. The closed field maze was constructed in a 8 × 14 m room. The arena floor was marked into 32 rectangles. Problem tasks were constructed using 2 m high plywood barriers. The path of the cow through the maze test was recorded by video. Cows solved six tests during 3 d. Tests 1 and 2 use a left-side solution, 3 and 4 a right-side solution, and 5 and 6 a central solution (Kilgour, 1981). Odd-numbered tests were in visual form, while even-numbered problems were non-visual. The motivation to finish the problem was access to a concentrate mix at the exit. Each test was performed twice (Fig. 1).

The cow was put into the maze entrance and a door closed behind it. The cow was timed from when it entered the maze until it got out. If the cow stood in the entrance for more than 3 min without moving, it was gently forced to move. If the cow stood at the end of the maze for more than 3 min without moving, it was led out. The cow was allowed to eat for only a few seconds, whereupon it was led out of the labyrinth to repeat the procedure. On the first observation day the cows completed five runs; the first run was for training.

Behavioural data were obtained by video observations and electronic measurements. The barn was equipped with video cameras for continuous filming of the cows' activities. There were computer techniques and software for evaluation (cameras: Samsung SCB-3000P, hard disk drive (HDD) recorder Versatile H.264 digital video recorder (DVR)) and the Observer XT Noldus (software for transmitting behavioural activities into numerical data).

### 2.7 Statistical analysis

The data were analysed using a general linear model ANOVA (Analysis of variance/Analysis of covariance, AOV/AOCV) by the statistical package STATISTIX, Version 10.0. The dependent variables were LBW, average daily gains (ADGs), milk performance, time taken to traverse the maze, and the number of vocalizations. The independent variables were treatment group and sire lineage.

The normality of data distribution was evaluated by the Wilk–Shapiro or Rankin plot procedure. The homogeneity of variance of the observed variables in groups was calculated by preliminary variance tests which determined whether the variabilities were equal. Bartlett's test for the equality of variance tests was used for an unequal size of samples. Differences between groups were tested by comparisons of mean ranks. Significant differences among means were tested by Bonferroni's test.

All values are reported as means ± standard error. The interactions between observed factors (treatment and sire lineage) were also computed.

The following model of general AOV/AOCV on observed factors (treatment and sire lineage) was used:

$$Y_{ij}=\mu +T_{i}+S_{j}+\alpha ij+\varepsilon ij,$$

where $Y_{ij}$ is a dependent variable, μ is the overall mean, $T_{i}$ is the effect of factor treatment on the level i, $S_{j}$ is the effect of factor sire lineage on the level j, $\alpha_{ij}$ is the interaction between factor T on the level i and factor S on the level j, and $\varepsilon_{ij}$ is the residual error.

**Figure 2:**
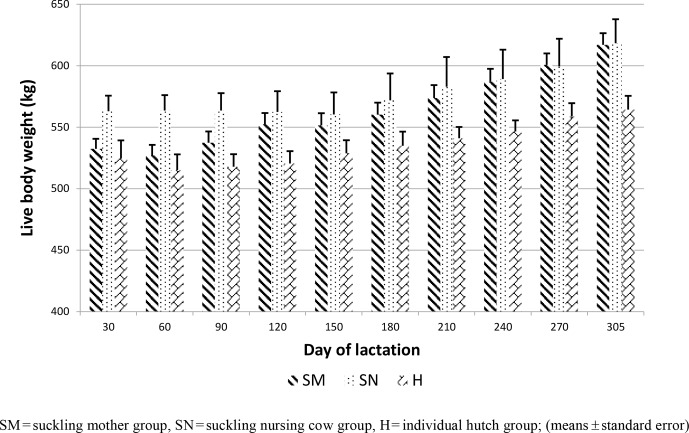
The course of growth of live body weight during lactation.

## 3 Results

### 3.1 Performance and health

At the 30th day, the LBW tended to be the highest in the SN group (SM 528.2 ± 11.4 kg, SN 571.7 ± 15.3 kg, H 533.2 ± 12.3 kg; P=0.0689). At the end of first lactation, at the 305 d, the highest LBW was in SN and the lowest in H (SM 612.6 ± 12.2 kg, SN 623.1 ± 16.4 kg, H 569.8 ± 13.2 kg; P=0.0165) (Fig. 2). The difference between SN and H groups was significant. No significant difference was found in the average daily gain from 30 to 305 d (SM 0.31 ± 0.06 kg, SN 0.18 ± 0.08 kg, H 0.13 ± 0.06 kg; P=0.1597) (Table 1).

**Table 1:** Growth and milk performance in the first lactation.

Factor		N	Live body weights at 30 d		Live body weights at 305 d	
			x ± SE	P value	x ± SE	P value
Group	1	13	528.25 ± 11.39	0.0689	612.64 ± 12.21${}^{\text{ab}}$	0.0165
	2	9	571.69 ± 15.35		623.12 ± 16.45${}^{\text{b}}$	
	3	13	533.19 ± 12.34		569.82 ± 13.22${}^{\text{a}}$	
Sire	1	4	549.39 ± 21.38	0.1863	595.93 ± 22.91	0.2793
	2	10	535.92 ± 13.05		596.68 ± 13.98	
	3	13	524.67 ± 11.53		587.47 ± 12.35	
	4	8	567.53 ± 15.03		627.37 ± 16.11	
Factor		N	Milk for 305 d (kg)		FCM for 305 d (kg)	
			x ± SE	P value	x ± SE	P value
Group	1	13	7143.9 ± 241.5	0.8459	6290.3 ± 203.2	0.7382
	2	9	7345.1 ± 319.0		6307.6 ± 268.4	
	3	13	7146.7 ± 187.9		6399.3 ± 197.1	
Sire	1	4	7201.4 ± 451.2	0.1507	6443.3 ± 379.7	0.0940
	2	10	7725.0 ± 252.7		6819.4 ± 212.7	
	3	13	7106.5 ± 241.7		6069.0 ± 203.3	
	4	8	6814.7 ± 294.8		6187.3 ± 248.1	

a, b – means with different letters are significant (p<0.05); Group 1 (SM: suckling mother); Group 2 (SN: suckling nursing cow); Group 3 (H: individual hutch); N: number of animals; SE: standard error.

According to the fathers, the LBW growth was not significantly different, but the daughters of S4 showed the highest LBW, both at the beginning and at the end of lactation (S1 549.4 ± 21.4 kg, S2 535.9 ± 13.0 kg, S3 524.7 ± 11.5 kg, S4 567.5 ± 15.3 kg; P=0.1863; S1 595.9 ± 22.9 kg, S2 596.7 ± 13.9 kg, S3 587.5 ± 12.3 kg, S4 627.4 ± 16.1 kg; P=0.2793) (Table 1).

The ages at the first calving were not different among treatment groups (SM 732.9 ± 15.5 d, SN 726.8 ± 9.2 d, H 763.1 ± 19.8 d; P=0.1798). Similarly, this indicator was not statistically different in comparison with sire lineages (S1 737.2 ± 31.5 d, S2 738.4 ± 11.1 d, S3 733.6 ± 19.2 d, S4 764.9 ± 22.3 d; P=0.4763). The two most important management variables relating to reproductive performance were not significantly different among groups (P=0.3107; P=0.4263). The first service interval (days between calving and first breeding) and open days were 65.5 ± 8.4 and 100.6 ± 17.2 d (SM); 71.9 ± 11.1 and 115.3 ± 20.4 d (SN); 83.5 ± 8.2 and 107.7 ± 18.2 d (H).

The SN group tended to have the highest milk yield (SM 7143.9 ± 241.5 kg, SN 7345.1 ± 319.0 kg, H 7146.7 ± 187.9 kg; P=0.8459), and the H group had the highest 3.5 % fat-corrected milk (FCM) (SM 6290.3 ± 203.2 kg, SN 6307.6 ± 268.4 kg, H 6399.3 ± 197.1 kg; P=0.7382) for 305 d of lactation.

The incidence of health problems was very low in all treatment groups, and there were no differences in the occurrence of illnesses in the study. Immediately after calving, two cows from the SN group for surgical calving (caesarean section) and one cow from the H group with a retained placenta (for increased risk of endometritis) were culled. However, these cows were not included in our evaluation. No cows were culled during first lactation in the experiment. Only cured cases of short-term health disorders were recorded: mild diarrhea (SM once, SN once), injury of the teat (SM once, SN once), injury of the hock with clinical lameness (H once), and mild mastitis with udder inflammation without systemic clinical signs (SM once, SN once, H once). No metabolic disorders, bronchopneumonia, or other respiratory diseases have been identified.

### 3.2 Behaviour

SN dairy cows ran a maze the fastest; this was clear in all tests. However, in the time taken to traverse the maze, significant differences among groups were noted only in Test 5 (SM 229.3 ± 25.0 s, SN 146.2 ± 32.3 s, H 205.6 ± 23.3 s; P=0.0441). Also, in the total time for all tests, group SN crossed the maze the fastest (SM 1141.4 ± 120.5 s, SN 810.3 ± 160.5 s, H 1120.8 ± 118.6 s; P=0.1233). We did not find any significant differences in the comparison of sires (Table 2).

**Table 2:** Maze behaviour in the fifth month of the first lactation.

Factor		N	Time taken to traverse		Total time taken to	
			the maze in Test 5		traverse the maze	
			x ± SE	P value	x ± SE	P value
Group	1	13	229.3 ± 25.0${}^{\text{a}}$	0.0441	1141.4 ± 120.5	0.1233
	2	9	146.2 ± 32.3${}^{\text{b}}$		810.3 ± 160.5	
	3	13	205.6 ± 23.5${}^{\text{ab}}$		1120.8 ± 118.6	
Sire	1	3	141.7 ± 27.3	0.1996	815.3 ± 32.9	0.5302
	2	10	186.4 ± 34.6		1012.6 ± 179.1	
	3	13	222.7 ± 25.8		1114.0 ± 124.6	
	4	6	183.0 ± 22.9		1037.3 ± 125.5	
Factor		N	Number vocalizations		Total number of	
			in Test 5		vocalizations	
			x ± SE	P value	x ± SE	P value
Group	1	13	6.2 ± 0.7${}^{\text{Aa}}$	0.0054	32.3 ± 5.7${}^{\text{A}}$	0.0019
	2	9	3.9 ± 1.0${}^{\text{b}}$		20.8 ± 4.4${}^{\text{AB}}$	
	3	13	2.9 ± 0.9${}^{\text{Bb}}$		9.9 ± 2.6${}^{\text{B}}$	
Sire	1	3	3.7 ± 0.9	0.0125	14.0 ± 4.6	0.3198
	2	10	6.3 ± 1.2${}^{\text{a}}$		24.4 ± 4.1	
	3	13	3.1 ± 0.6${}^{\text{b}}$		16.1 ± 4.9	
	4	6	3.5 ± 1.2${}^{\text{b}}$		23.8 ± 8.5	

Interactions: group × sire (P=0.0498) Interactions: group × sire (P=0.0471). a, b – means with different letters are significant (p<0.05); A, B – means with different letters are significant (p<0.01); Group 1 (SM: suckling mother); Group 2 (SN: suckling nursing cow); Group 3 (H: individual hutch); N: number of animals; SE: standard error.

In all tests, SM cows vocalized the most and H cows the least. The total vocalization number for all tests differed significantly (SM 32.3 ± 5.7, SN 20.8  ± 4.4, H 9.9  ± 2.6; P=0.0019). Except for the sixth test (P=0.0611) and the total number of vocalizations for all tests (P=0.3198), there were significant differences in the number of moos when comparing the offspring of different sires. The daughters of Sire 2 had the most vocalizations. Interactions between group and sire factors were calculated in the fifth test, including for total vocalization number (P=0.0498; P=0.0471) (Table 2).

## 4 Discussion

### 4.1 Performance

The trend of the highest LBW of the SN group was shown at the beginning of lactation. The most intense growth of this group was maintained until the end of the experiment.

We assume it was caused by the influence of rearing on weaning from the milk-fed period. According to our previous study (Broucek et al., 2020), from the 4th to the 84th day the calves of the SM group received 406.4 ± 48.23 kg (5.08 kg/d) milk, the SN group had 412.52 ± 42.93 kg (5.16 kg/d) milk, and the calves of the H group had 408.44 ± 32.65 kg (5.11 kg/d) of MR. We also had similar results in the current study. From the 4th to the 84th day, the heifers of the SM group had an intake of 407.15 ± 10.73 kg (5.09 kg/d) milk, the SN group received 414.02 ± 8.92 kg (5.17 kg/d) milk, and the calves of the H group had 408.12 ± 9.12 kg (5.10 kg/d) of MR.

Suckling time at the mother's udder (10 min) of SM calves was determined according to Passillé de and Rushen (2006). The calves were weighed immediately before and after each suckling to assess the milk intake. For three times 10 min, the calf should receive 6 kg of milk from the average herd cow. The number of SN calves per nursing cow was determined according to the milk yield of selected cows, so that 6 kg of milk per calf and day was available. According to older work (Broucek et al., 1995), it is possible that the SN heifer calves consumed a higher amount of milk than the estimated 6 kg/d. A cow which is stimulated by frequent suckling can produce more milk. However, all three treatment groups received a maximum of 6 kg of milk or MR daily.

Previous experiments have shown that calves that were suckled by their mothers or foster cows during the milk-feeding period achieved a higher ADG than calves separated from their dams at birth, probably due to the higher milk intake of these naturally reared calves (Krohn et al., 1999; Bar-Peled et al., 1997). Generally, feeding high levels of milk can improve heifer performance (Grøndahl et al., 2007; Moallem et al., 2010; Soberon et al., 2012; Asheim et al., 2016; Mala et al., 2019). These results support our findings that the increased LBW in SN cows was a result of greater milk intake to weaning. A lower LBW at the 30th day in the SM group can be explained as an effect of lower milk intake (Mejia et al., 1998; Fröberg et al., 2007; Asheim et al., 2016).

The significantly highest intake of starter concentrate mixture was recorded in group SN (39.2 kg) and the lowest in group SM (34.2 kg). No significant differences were found in alfalfa hay consumption (Broucek et al., 2020). In our opinion, the higher growth in SN heifers was caused by the suckling of more milk than was calculated, but also by faster habitude of calves to solid feed and higher solid feed intake by social facilitation. We can assume a higher amount of sucked milk in the SN group to weaning than measured by milk yield control. The calf is likely to suck out more milk from the nursing cow's udder than is obtained during milking and, on the other hand, a cow stimulated by suction produces more milk. Calves fed ad libitum by suckling are able to drink more than 10 kg per day (Davis and Drackley, 1998). During the second week of life, Appleby et al. (2001) recorded 8.4 kg/d milk and 9.76 kg/d milk in the fourth week. Kiezebrink et al. (2015) limited the amount of milk to 8 L per day and by weaning in the eighth week, the calves reached an intake of 399.1 kg.

Suckling of several calves empties the udder properly and can increase milk creation. Also, suckling of milk from the udder increases the level of growth hormone (Lupoli et al., 2000; Fröberg et al., 2008). Heifers fed whole milk were heavier than those fed milk replacer, probably because of better bioavailability of nutrients (Lee et al., 2009).

There was also a significantly higher body weight at 305 d in the SN group compared to group H. The reason was again the rearing manner and the large weight difference in favour of the SN group (571.69 kg versus 533.19 kg) at the beginning of lactation. The heifers of the SN group probably received more valuable liquid nutrition from udders (whole milk) than the animals from group H (milk replacer). SN cows were kept in loose group housing from the fourth day of life, the longest of all monitored groups (Broucek et al., 2020), and group housing may also stimulate appetite (Yanar et al., 2000; Wójcik et al., 2013).

The majority of studies have reported that the benefits for growth to weaning were maintained for months after separation (Khattak et al., 2018; Meagher et al., 2019). The heifers which were provided with milk for a longer time and weaned late showed higher LBW (Kisac et al., 2011; Miller-Cushon et al., 2013).

Some studies report reduced growth in suckled calves, particularly in the weeks immediately after weaning. These results were likely due to the challenge of weaning calves from high volumes of milk, while most artificially reared calves in these studies were fed restricted volumes (Uys, 2008; Fröberg et al., 2011; Novak et al., 2019). The separation and weaning can be concurrent. Johnsen et al. (2015b) showed that if calves can be separated and weaned in time, the decline in growth is lower. According to Conneely et al. (2014), reduced growth following weaning in calves fed higher quantities of milk before weaning occurs because the high milk intake depresses concentrate mixture consumption. The present work showed that the different intake of milk drink in weaning could be reflected in the LBW and milk production of monitored cows in adulthood.

Beaver et al. (2019) showed, in their systematic review, that literature on calf health does not indicate that early separation is advantageous. Authors Haley et al. (2005), Loberg et al. (2007), Loberg et al. (2008), and Johnsen et al. (2016) point out that the process of weaning poses more difficulties in dam rearing due to breaking the strong bond between mother and young. According to the review of Kälber and Barth (2014), it seems that each weaning strategy (at birth, gradually, weaning in two steps, auditory and visual contact between dam and young after separation) is always associated with stress in calves. Scoley et al. (2019) compared gradual and abrupt (with complete withdrawal of milk) methods of weaning and they did not find significant impact on calf live weight. Their study suggested that gradual weaning of calves may lead to a more prolonged sense of frustration than that experienced by abruptly weaned calves.

There was no difference among sires in LBW at 30 or 305 d of lactation, but the cows descended from S4 were heavier than the other sire lineage groups. De la Cruz-Cruz et al. (2019) found that artificial rearing of calves presents a combination of emotional and nutritional stress that reduces their immune response and can alter their genetic growth premise.

Group SN displayed a tendency for the highest production of milk, and the H group displayed this for FCM for 305 d lactation. Potential mechanisms for this increase in production have been suggested, such as improved mammary gland development (Brown et al., 2005; Daniels et al., 2009; Morrison et al., 2012; de la Cruz-Cruz et al., 2019); the keys to explaining the differences in milk production are the heifer feeding to weaning, growth during rearing, and LBW after calving. It is difficult to explain this phenomenon of non-significant increase in FCM production in group H. This could be due to the higher milk fat content in weaning, and therefore these cows had a higher FCM. Shamay et al. (2005), Moallem et al. (2010), and Chester-Jones et al. (2017) found that milk-fed calves had higher daily average of fat-corrected milk (P<0.01) during the first lactation. Kiezebrink et al. (2015) fed calves 8 L versus 4 L whole milk/d but found no differences in first-lactation performance. However, the H group was fed by MR and not milk.

More articles found positive effects between the level of liquid nutrition during the milk-fed period and following milk yield production (Bar-Peled et al., 1997; Shamay et al., 2005). Moallem et al. (2010) reported that heifers fed whole milk produced 10 % more milk than heifers fed milk replacer. There are also indications that early high milk intake or improved nutrition early in life in heifers increased a milk yield in primiparous cows (Shamay et al., 2005; Drackley et al., 2008). However, the results of Davis Rincker et al. (2011) and Kiezebrink et al. (2015) confirm that enhanced whole-milk feeding did not affect post-calving LBW, or 305 d milk yield in the first lactation.

A high weaning LBW may result in a higher LBW at calving. Authors Langhout and Wagenaar (2006), Terré et al. (2009), Khan et al. (2011), and Asheim et al. (2016) found that a high live body weight for heifers at calving had a positive effect on milk yield in the first lactation.

Genetic and environmental influences of the sire on milk production are known and have been well documented (Hayes et al., 2003). The sire lineage influences a large part of the population so its genetic qualities are effective as a stabilization factor. According to Coffey et al. (2006), growth in Holstein dairy heifers has been significantly altered in line with selection, primarily for yield. This alteration might have consequences in later life for important traits such as the fertility and milk yield. However, the effect of paternal origin has not been proven in the assessment of LBW growth nor milk performance.

### 4.2 Behaviour

The shortest time of running across the maze was recorded in the SN group. How can this be explained? In foster cow rearing systems (such as SN) calves have to compete with other calves, and this can have an effect on their behaviour after weaning or calving. Maternal care and social contact also played an important role. On the other hand, SM calves were separated from their dam after 3 weeks and then had to get used to bucket feeding. This could cause a relevant level of stress in the calves that also affects their behaviour during or after this change.

The results of rearing influence also suggest that providing enrichment in the form of a foster teat during the milk feeding period can change calf's behaviour responses in ethological tests. Calves of the SN group took less time to find the reward during the learning tests. Calves housed in unenriched environments (H) or enriched environment for 21 d only (SM) had lower flexibility in the maze.

Purcell and Arave (1991) found that pre-weaning isolation affected learning ability. Also, in the studies of Gaillard et al. (2014) and Meagher et al. (2016), the individually housed calves had learning deficits versus paired or grouped calves. We assume that cows reared in individual hutches cannot sufficiently express their social behaviour; they cannot quickly cope with the new situation, and therefore have impaired learning abilities. These results confirm the previous findings of Costa et al. (2016). Wagner et al. (2012) reported that dam-reared heifers transitioned better into the lactating herd, suggesting that social housing of heifers may enhance social skills that are useful later in life. Group housing of calves is associated with increased LBW gains during the milk feeding period and after weaning compared with individual housing, likely due to increased dry-matter intake (Warnick et al., 1977; Jensen et al., 2015).

Latham and Mason (2008) wrote that animals with maternal deprivation are less able to cope in a low-stress manner with normal social interactions with conspecifics. Flower and Weary (2001) reported that calves kept with their mother for 14 d exhibited more intense social behaviour towards unfamiliar calves. Heifers reared on a foster cow were socially more active and had clearer social structures relative to individually reared heifers (Le Neindre and Sourd, 1984).

Meagher et al. (2016) found that social rearing, and especially dam rearing, improved the calves' ability to learn as compared to calves from individual rearing. They wrote that whether these social skills and learning abilities are maintained in adult cows is not yet known. When tested in isolation, dam-reared cows in comparison to conventionally reared cows tended to show more locomotion and exploratory behaviour (Le Neindre, 1989; Kälber and Barth, 2014) and be more active (Wagner et al., 2015).

Costa et al. (2016) reviewed the articles which examined the relationship between the social environment and behaviour in calves. They found that socially reared calves are less fearful and more dominant when mixed in groups later in life compared with calves that have been reared in isolation. Socially reared calves had a higher preference toward an unknown food than calves reared individually (Costa et al., 2014). According to Jensen and Larsen (2014), calves housed individually or with only limited contact were more fearful than pair-housed calves. These reports suggest that social contact with peers is important for the calf. Dairy cows that had experienced 12 weeks of contact with the dam showed higher behavioural activity during the isolation test than cows that had been individually raised (Wagner et al., 2015).

Dairy cattle can probably be preconditioned to stressful situations. However, if such preconditioning to psychological stresses is to be achieved, farm animals must have the chances to learn and remember. Social behaviour and bonds with the mother are absent in artificial rearing systems (such as housing in hutches), leaving these systems different in terms of calf welfare. Social isolation early in life can impair cognition in animals (Costa et al., 2016). The calf also learns, as do other animals, the features of the species to which it will later direct its innate sexual responses (Kilgour et al., 1981; Arave et al., 1992; Veissier, 1993). However, extended cow–calf contact aggravates the acute distress responses, but it can have positive effects on behaviours relevant to welfare in the longer term. Prolonged periods with nursing cow contact may provide longer-term benefits for later cow behavioural development (Meagher et al., 2019).

The barn housing area may not suit all animals; their welfare deteriorates. Dairy cows in particular must be able to adapt quickly. When animals are introduced into new a housing system, they have to learn how the resources are distributed within it. At first sight, such a differentiation in the use of different areas of a housing system for different activities seems to be trivial. This is very similar to entering a maze. The size and nature of the object in which the animal has become interested determines the speed of approach (Fraser and Broom, 1997; Lauber et al., 2009). The speed of an animal in running through various types of labyrinths has been used as a measure of animal intelligence and learning ability for a long time (Kilgour, 1987; Arave, 1996).

In recent years, dairy cattle have been increasingly used in experiments on discrimination learning or spatial learning. These studies show that farm animals are able to learn difficult experimental tasks (Broucek et al., 2003; Wechsler and Lea, 2007; Büscher and Quinckhardt, 2009; Manteuffel et al., 2009).

Overall, the results of this experiment suggest that sire lineage has little or no effect on the responses of dairy cows in the various tests used. There are many reports of variation in fear- and anxiety-related behaviour in cattle, which may be partly genetically determined. Hohenboken (1987) suggests that we might use the knowledge of such genetic variation in behaviour to improve animal welfare (Wredle et al., 2004).

SM cows vocalized the most and H cows the least. It is obvious that SM cows were not only the slowest in solving tests, but they vocalized the most. This was certainly their manifestation of fear of an unknown environment. Mooing can be considered their fear-related responses (Boissy, 1995).

The vocalization was probably related to the time point when calves were separated from their mothers or nursing cows. Vocalization behaviour increases with longer contact with the mother from birth (Johnsen et al., 2015a; Stehulova et al., 2017; Steele, 2019). We must point out an important psychological factor that affects dairy cows: their independence. When we compare the groups SM and H, it is obvious that the separation time was very different. The heifers of the H group did not actually form a bond with the mother, but the heifers in the SM group had to be closely dependent on the mother. Also, it is likely that the vocal response emphasizes how cattle have a level of habituation to social isolation (Mueller and Schrader, 2005; Siebert et al., 2011; Juhas and Strapak, 2013; Green et al., 2018).

In the current study, significant differences in the number of moos among sire lineage groups were found. The daughters of Sire 2 showed the most vocalizations during all the tests. The significant interaction between treatment and sire lineage in the total number of mooings may indicate that groups according to sires have opposite reactions across treatment groups. We can state that sire lineage modified some of the responses to the maze tests.

The genetic impact on behaviour is not direct but results from a complex response network of neurophysiological and structural factors, like hormones and proteins, themselves products of indirect genetic effects (Johnston and Edwards, 2002). Breeding for cattle behaviour has been intensively discussed (reviewed in Friedrich et al., 2015). Increasing attention has been paid to cattle temperament for its benefit to working safety, adaptability to new housing conditions, animal welfare, and production. In some countries, the milking temperament of dairy cattle is already integrated into breeding programmes as a selection index.

## 5 Conclusions

Many of environmentally conscious people and experienced dairy farmers are not satisfied with the artificial feeding system during the rearing of heifers. In order to improve the welfare of their dairy cattle, a number of organic farms introduced suckling systems. These systems make better use of the growing potential of calves in the first months of their lives.

The purpose of the present study was to find whether dairy cow growth and milk performance and behaviour are affected by their rearing during the milk-fed period and by the sire lineage. Three treatments were used: SM, with mother to the 21st day, then group pen; SN, with nursing cow; H, in hutch to the 56th day, then group pen.

The most intense growth and milk yield of the SN group was maintained from the beginning until the end of the first lactation. Group SN crossed the maze the fastest. In the evaluation of all maze tests, group SN appears to be the most adaptable, and cows from group SM were the least adaptable.

The results indicated that the rearing method to weaning may have an impact on dairy cow performance and behaviour. The sire lineage influenced the responses to the maze tests only.

According to our results, it is possible to successfully use the method of heifer rearing with the help of nursing (foster) cows in dairy farming. These management systems can be a viable option for some producers even in our modern dairy systems.

## Data Availability

No data sets were used in this article.
